# Comprehensive analysis of single-cell and bulk transcriptomes reveals key B-cell genes and immune microenvironment regulation in bladder cancer

**DOI:** 10.3389/fimmu.2025.1600254

**Published:** 2025-10-17

**Authors:** Lijun Wang, Juan Yang, Zhangxiao Xu, Bo Tao, Yunpeng He, Yuan Zhao, Jian Wu, Yiran Ma, Zitao Zhong, Lin Ye

**Affiliations:** ^1^ Faculty of Life Science and Technology & The Affiliated Anning First People’s Hospital, Kunming University of Science and Technology, Kunming, China; ^2^ Faculty of Life Science and Technology, Kunming University of Science and Technology, Kunming, Yunnan, China

**Keywords:** bladder cancer, B cells, immune microenvironment, single-cell RNA-seq analysis, biomarkers, RT-qPCR

## Abstract

**Background:**

Bladder cancer is a significant malignancy, for which prognostic prediction and understanding of the tumor immune microenvironment are crucial. B cells play a key regulatory role in this environment, making their study essential for advancing bladder cancer research.

**Methods:**

In this study, a multi-omics analysis strategy combining single-cell RNA sequencing (scRNA-seq) and bulk RNA-seq was used to establish single-cell transcriptome profiles of tumor tissues from bladder cancer patients, focusing on B-cell populations and their interactions with other cell types in the tumor microenvironment. Large public databases were used to screen for key prognostic genes associated with bladder cancer B cells, and their biomarker expression was verified by *in vitro* experiments.

**Results:**

Based on tumor samples from eight patients with bladder cancer and four normal samples, we selected 84, 967 cells for single-cell sequencing analysis. From these, we identified 10, 967 B cells and identified 508 key genes associated with B cells in bladder cancer from five different B cell subtypes. By integrating a large amount of RNA sequencing data, we identified *VCL*, *FLNA*, *TAGLN*, *ACTA2*, *COL6A2*, and *CALD1* as potential biomarkers for B-cell-associated bladder cancer, and experimentally verified that these markers were significantly lower in bladder cancer patients than in the normal group, and were effective in predicting the survival rate of the patients and the status of the tumor immune microenvironment.

**Conclusions:**

Using a combination of transcriptomic and experimental validation at single-cell and batch levels, this study provides insights into the key gene signatures of B cells from patients with bladder cancer and their roles in regulating the tumor immune microenvironment, providing new biomarkers and potential therapeutic targets for predicting patient’ prognosis and immunotherapy response.

## 1 Introduction

Bladder cancer (BCa) is a malignant tumor originating from the epithelium of the urinary tract, which continues to increase in morbidity and mortality, and is one of the most common malignant tumors (1). Epidemiological estimates for 2025 indicate that bladder cancer is the fourth most common cancer among men in the United States (n = 65,080), with a total of 84,870 new cases nationwide. The incidence in men is 3.3 times higher than that in women, showing a significant difference (2). Bladder cancer is a highly heterogeneous disease, with many challenges in its classification, staging, and grading (3, 4). Bladder cancer is characterized by high rates of recurrence and metastasis. Based on the depth of tumor infiltration, they can be divided into non-muscle invasive bladder cancer (NMIBC) and muscle invasive bladder cancer (MIBC) (5). Of these, 75% of uroepithelial carcinomas of the bladder present as non-muscle invasive bladder cancer (6), and the other 25% of urothelial carcinomas of the bladder present as muscle-invasive bladder cancer with a risk of metastatic spread, with a 5-year survival rate of approximately 40%-50% (7), approximately 50% of bladder cancer cases with muscle invasive bladder cancer eventually metastasize, and the 5-year survival rate for muscle invasive bladder cancer with distant metastases is only 10% (8), which is the main cause of death in bladder cancer patients. Currently, radical cystectomy (RC) combined with chemotherapy or immunotherapy is the first-line treatment for BCa (9). Although chemotherapy and immunotherapy can improve survival to some extent, a subset of patients respond poorly to these therapies, resulting in missed opportunities for RC and reduced survival (10, 11). However, in some cases, it may not be effective in preventing cancer recurrence and metastasis (12). Therefore, recurrence, metastasis, and spread of cancer have become one of the biggest resistances to cancer treatment, and it is extremely important to study the mechanism of bladder cancer development, metastasis, and spread, and to find new targets for bladder cancer treatment.

The tumor microenvironment (TME) has been a hotspot in cancer biology research and is a relevant therapeutic target for drug discovery. Notably bladder cancer is one of the cancers with the least immune infiltration (13), This may account for the poor response to anti-PD1 therapy. Molecular subtypes of bladder cancer show different cell type-specific expression patterns (14), suggesting that the heterogeneity of BCa is, at least in part, due to the different cell type components of the microenvironment. However, until recently, relevant studies have been scarce. Previous studies have shown that the abundance of B cells in cancer is positively correlated with a favorable clinical outcome, whereas others have shown that they promote tumors, implying that the biological function of B cells is a complex landscape (15). Relatively little is known about comprehensive molecular analyses of B cells in bladder cancer. Therefore, the biological functions of B cells in bladder cancer remain to be explored.

**Figure 1:**
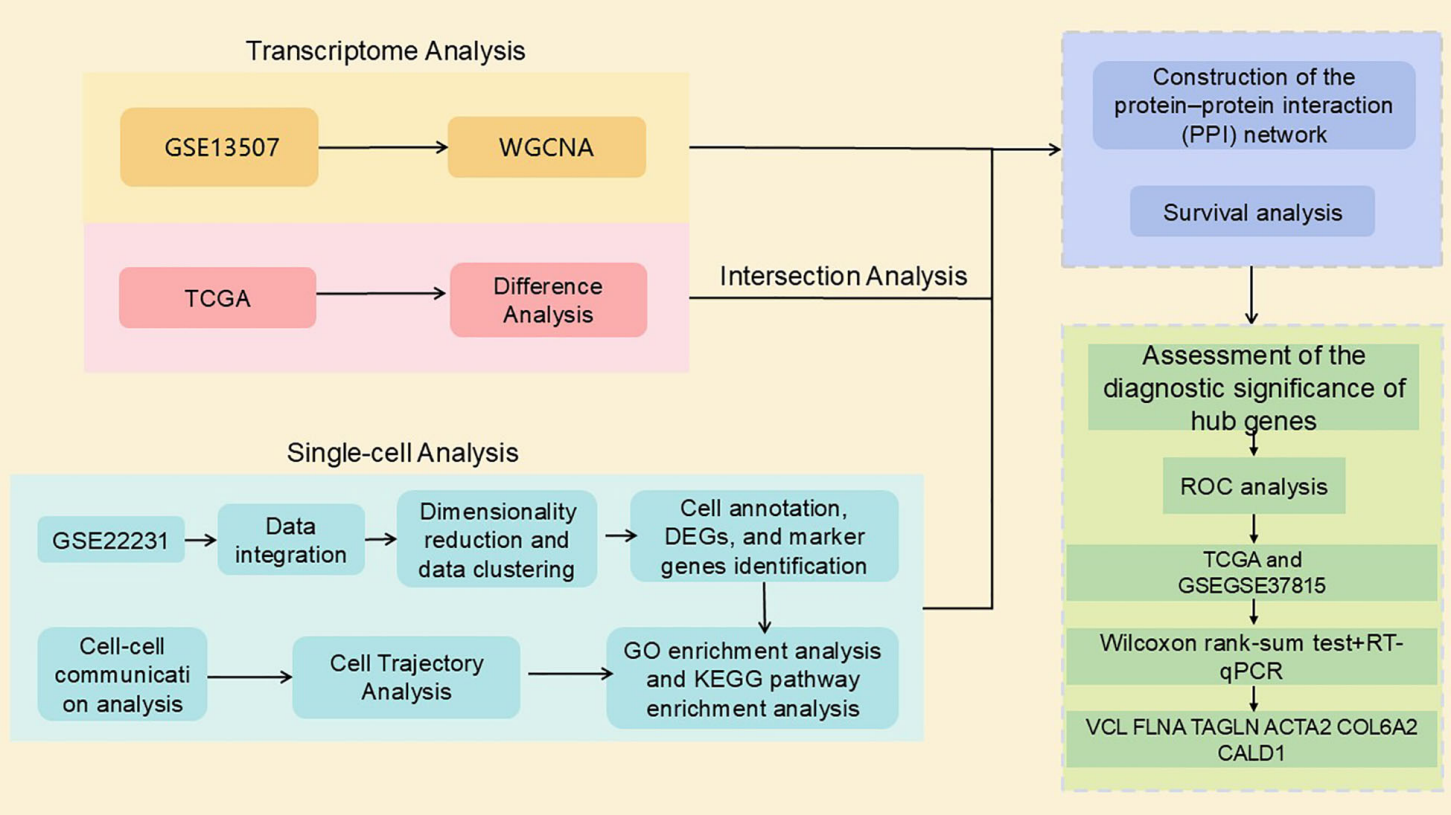
Research design.

The rise in single-cell RNA sequencing (scRNA-seq) technology has provided an unprecedented opportunity to resolve the molecular signatures of different immune cell populations in the TME (16). The scRNA-seq provides more precise and detailed analyses at the single-cell level than traditional bulk RNA-sequencing methods (17). In this study, we innovatively combined bulk RNA seq and scRNA-seq data to systematically reveal the infiltration pattern of B cells in the microenvironment of bladder cancer, and successfully identified seven bladder cancer markers with potential clinical applications. In this study, the molecular features of tumor-infiltrating B cells were explored in depth and their specific marker genes were identified through single-cell RNA sequencing analysis of bladder cancer samples. RNA sequencing data from large databases were integrated and analyzed to screen and validate potential key genes associated with cancer immune responses. The workflow of this study is illustrated in **Figure 1**.

## 2 Methods

### 2.1 Data source and preprocessing

In this study, a multi-omics integration analysis strategy was used to integrate four independent datasets from The Cancer Genome Atlas (TCGA) (https://www.cancer.gov/ccg/research/genome-sequencing/tcga) bladder cancer dataset, and the Comprehensive Gene Expression Database (GEO) (https://www.ncbi.nlm.nih.gov/) (**Table 1**). Eight bladder cancer tissue samples and four normal tissue samples from nine patients in the GSE222315 dataset were selected for the scRNA-seq study. The selection criteria were as follows:(a) each sample should contain no less than 300 and no more than 7000 cells; (b) each cell should express more than 300 (250) genes; (c) each gene should be expressed in at least three cells; and (d) the mitochondrial RNA content in each cell should be less than 20% (18). Finally, we screened using the R package Seurat and obtained 84, 967 cells for subsequent analysis. Differentially expressed genes (DEGs) were identified from TCGA bladder cancer dataset using the DESeq, limma, and Wilcoxon function packages of the R package. Specifically, we applied a threshold of |log2 FoldChange| > 2 with an adjusted *P* < 0.05 to select DEGs for subsequent analysis (19). The GSE37815 dataset was used for marker gene validation.

**Table 1:** Grouping information.

Database	Dataset	Grouping information		Purpose
		Experimental group	Control group	
TCGA	431 sample	412	19	Differential Analysis
GEO	GSE222315	8	4	Single-cell Transcriptome Analysis
	GSE13507	187	9	WGCNA
	GSE37815	18	6	Validation Set

### 2.2 Data integration

After data integration and filtering, the Seurat package was used for data normalization and integration analyses. First, the gene expression values were normalized using the ‘NormalizeData’ function. Specifically, the expression value of each gene was multiplied by the total gene expression of the cell, and the scaling factor (10,000) and the natural logarithmic transformation (log(x+1)) were performed to avoid a zero logarithm. Next, 2000–3000 highly variable genes were identified by the ‘FindVariableFeatures’ function and centered using the ‘ScaleData’ function. To eliminate technical differences between batches of samples (due to the inability to sequence samples simultaneously), we used the ‘FindIntegrationAnchors’ or ‘RunHarmony’ function to set up 2000 anchors for data integration. This anchor-based integration strategy can effectively map homologous cell types from different datasets to a small number of key anchors, thereby reducing the batch effect and improving the comparability and reliability of data.

### 2.3 Dimensionality reduction and data clustering

Dimensionality Reduction and Data Clustering: Considering that each gene in the sample exists as a dimension, high-dimensional data becomes difficult to visualize. Therefore, dimensionality reduction techniques must be employed to represent the true data structure by using a reduced number of dimensions (20). First, the highly variable set of genes obtained was downscaled using the ‘RunPCA’ function in the Seurat package. Subsequently, data integration was performed by setting 2000 anchors using the ‘FindIntegrationAnchors’ (or ‘RunHarmony’) function. This ‘anchor’ based integration strategy effectively converts the same cell types from different datasets into a small number of key anchors, thus mitigating batch effects. Next, the ‘FindNeighbors’ and ‘FindClusters’ functions in the Seurat package were used to perform cluster analysis of the downscaled data. To enhance the visual presentation of the clustering results, the t-distributed stochastic nearest neighbor embedding (t-SNE) method was applied. Specifically, a clear visualization of the cell population was achieved by the ‘RunTSNE’ function of the Seurat package with the parameters dims=1:15 and resolution=1.0.

### 2.4 Cell annotation, DEGs, and marker genes identification

Cellular annotation, DEG and marker gene identification were performed by reviewing relevant literature and websites and manually searching for cellular marker genes for cell-type annotation. This approach effectively correlates gene expression in different cell types with that in cells. The weakest correlation for each cell type was eliminated through iterations and the corresponding cell types were identified (21). In this study, samples were manually annotated for cell types using literature and web resources. Subsequently, DEG were identified using the ‘FindAllMarkers’ function in the Seurat software package. A threshold value of |log2FoldChange| > 2 (0.25 between cells and between groups) was set and adjusted to *P* < 0.05. Finally, marker genes specific to each cell type were identified using the ‘FindAllMarkers’ function of the Seurat software package. This series of analyses provides an important basis for precise annotation and functional study of cell types.

### 2.5 GO enrichment analysis and KEGG pathway enrichment analysis

Gene Ontology (GO) and Kyoto Encyclopedia of Genes and Genomes (KEGG) pathway enrichment analyses were performed using the David and Metascape databases (clusterProfler packages, version 3.14.3). The analysis revealed functional enrichment of genes and related signalling pathways. All the results were visualized using the ggplot2 software package (22, 23).

### 2.6 Cell trajectory analysis

Cells constantly undergo dynamic changes, transforming from one cell type to another, which leads to changes in gene expression and functional state (24). Each cell was arranged along a corresponding cell track, representing a pseudotemporal order, and cells were grouped into different differentiated states by employing gene expression profiles. We used the Monocle2 package for the pseudotemporal analysis of B-cell subtypes (25). To explore the differentiation trajectories of B cell subtypes and related genes in different states, we used the ‘plot_cell_trajectory’ function to sort the cells according to their pseudotimes. The ‘BEAM’ function was used to identify genes responsible for cell branching and differentiation. The ‘plot_genes_branched_heatmap’ function was used to visualize the results.

### 2.7 Cell-cell communication analysis

The analysis of cell-cell communication is based on the interaction of ligands and receptors on the cell surface, and this intercellular communication plays a key role in a variety of biological processes. To investigate the interaction patterns between different cell types, the CellChat software package was used to construct cellular communication networks. The software simulates the communication between cells by constructing an interaction network of ligands, receptors, and their related factors. Based on the gene expression profiles of ligands and receptors in different cell types, CellChat was able to infer the strength of cellular interactions and reveal the rich patterns of ligand-receptor interactions between different cell types. This analysis provides an important basis for a deeper understanding of intercellular signalling and functional regulation (26).

### 2.8 Construction of weighted gene co-expression network

Weighted gene co-expression networks were constructed using the R package ‘WGCNA’ (27). Sample analyses were initiated by screening for outlier samples using clustering methods. Subsequently, the gene expression matrix was converted into a similarity matrix by calculating the Pearson correlation value (cmn) and adjacency (amn) between genes, where parameter *β* served as a soft threshold that could modulate the correlation between genes. Based on the assessment of scale independence and average connectivity, the soft threshold power was set to 8 (*β* = 8, R^2^ = 0.9). The neighbor-joining matrix was further transformed into a topological overlap matrix so that the gene interactions conformed maximally to the scale-free distribution characteristics. Hierarchical clustering was performed using the dynamic tree-cutting algorithm with the unsigned network construction type, which was set to two to determine the module division, and the minimum module size of the gene dendrograms was set to 30. The module difference degree (MES) of the module dendrograms was calculated, and modules with a difference degree of less than 0.3 were merged to construct the final network structure.

### 2.9 Differentially expressed genes for bladder cancer-related genes

412 bladder cancer gene expression data and 19 paraneoplastic tissue gene datasets were downloaded from TCGA database. The downloaded gene datasets were integrated into a matrix using Perl (command line) software. Screening criteria for identifying DEGs were established using the R packages DESeq, limma, and Wilcoxon. Specifically, we applied a threshold of |log2 fold change | > 2 with an adjusted *P* < 0.05. The DEGs were selected for subsequent analyses. A Venn diagram was used to analyze the intersection of three sets of gene sets: sc-seq tagged genes from bladder cancer-associated B cells, high ESTIMATEScore-related genes obtained from WGCNA analysis, and differentially expressed genes screened by the TCGA database using the three methods to identify core overlapping genes.

### 2.10 Integrated survival, ROC, and PPI network analyses for the identification of bladder cancer biomarkers

To select the core genes with prognostic value, Kaplan-Meier survival analysis was performed on these key genes using the R software package ‘survivalPath’ (28), and survival differences were evaluated using the log-rank test. Genes with *P* < 0.05 were considered to have prognostic significance and were subjected to further in-depth analysis. Assessment of the diagnostic significance of hub genes. To investigate whether prognostic genes could distinguish tumor samples from non-tumor samples, we used the R package ‘survival ROC’ (29) and ROC analyses were performed on the hub genes. To elucidate the interaction network of infiltrating immune cell-associated genes at the protein level, protein-protein interaction (PPI) network mapping was constructed using the STRING database (https://string-db.org/). The interactions were visualized using the Cytoscape 3.7.1 platform to clearly demonstrate the functional association patterns among the genes.

### 2.11 Core gene database and protein validation

The expression levels of the core genes in tumor and non-tumor samples were compared in TCGA and GSE37815 datasets. Additionally, the protein expression of these genes in bladder cancer was validated using the Human Protein Atlas (HPA) database (https://www.proteinatlas.org/).

### 2.12 Validation of core genes in cells and clinical samples by RT-qPCR

A normal human uroepithelial cell line (SV-HUC-1) and three human bladder cancer cell lines (5637, T24, and HT1376) were obtained from Yunnan Tengyue Biotech Co. E2112), 5637 cells, and HT1376 cells were cultured in RPMI-1640 medium (Gibco, C11875500BT), T24 cells were cultured in McCoy’s 5A medium (EvaCell, E2110), and the above cells were cultured in medium supplemented with 10% FBS (Gibco, A5256701) and 1% penicillin-streptomycin (Gibco,15140122). Cells were cultured at 37 °C, 5% CO2 cell culture incubator. Total RNA was extracted using the RNAfast200 kit (Shanghai Feijie Biotechnology Co., Ltd.), and mRNA was reverse-transcribed into cDNA using the Evo M-MLV Reverse Transcription Reagent Pre-mix (Hunan Acres Bio, AG11706). RT-qPCR was performed using the SYBR Green Pro Taq Hs Pre-mixed qPCR Kit (Hunan Acres Bio, AG11701). RT-qPCR was performed to verify the expression of the biomarkers in normal human uroepithelial cells (SV-HUC-1) and human bladder cancer cell lines (5637, T24, and HT1376). To further validate the experimental results, we also collected tumor tissues and adjacent normal tissues from seven bladder cancer patients at the First People’s Hospital of Anning, Affiliated Hospital of Kunming University of Science and Technology. All participants provided written informed consent, and the study was approved by the Ethics Committee of the First People’s Hospital of Anning, Affiliated Hospital of Kunming University of Science and Technology. The sequences of all primers are listed in **Table 2**.

**Table 2:** Primers of hub genes.

Genes	Forword	Reverse
VCL	CTGCAGACCAAAACCAACCG	GGTCACACTTGGCGAGAAGA
FLNA	CTGCTCGGTCGAGTACATCC	TCACATCATGCACAGGGACC
TAGLN	AGGTCTGGCTGAAGAATGGC	CACCTGCTCCATCTGCTTGA
ACTA2	ACTGCCTTGGTGTGTGACAA	TCCCAGTTGGTGATGATGCC
COL6A2	AGCCTACGGAGAGTGCTACA	AGCTTGCCCTTCTGTCCATC
CALD1	TTTGAGCGTCGCAGAGAACT	TTCTGGGCATTCACCTCCAC
GADPH	CAGGAGGCATTGCTGATGAT	GAAGGCTGGGGCTCATTT

### 2.13 Data processing

Statistical analysis was performed using the Statistical Analysis R software (version 4.4.3) and GraphPad Prism 9.0 (GraphPad Software, USA). Differences between samples were assessed using the Wilcoxon rank-sum test, and statistical significance was determined using Student’s t-test. Experimental results are expressed as the mean ± standard deviation (mean ± SD), with all data derived from at least three independent experiments. Significance was denoted as follows: NS: p > 0.05, *: p < 0.05, **: p < 0.01, ***: p < 0.001, and ****: p < 0.0001.

## 3 Results

### 3.1 Single-cell transcriptome profiles of bladder cancer

**Figure 2:**
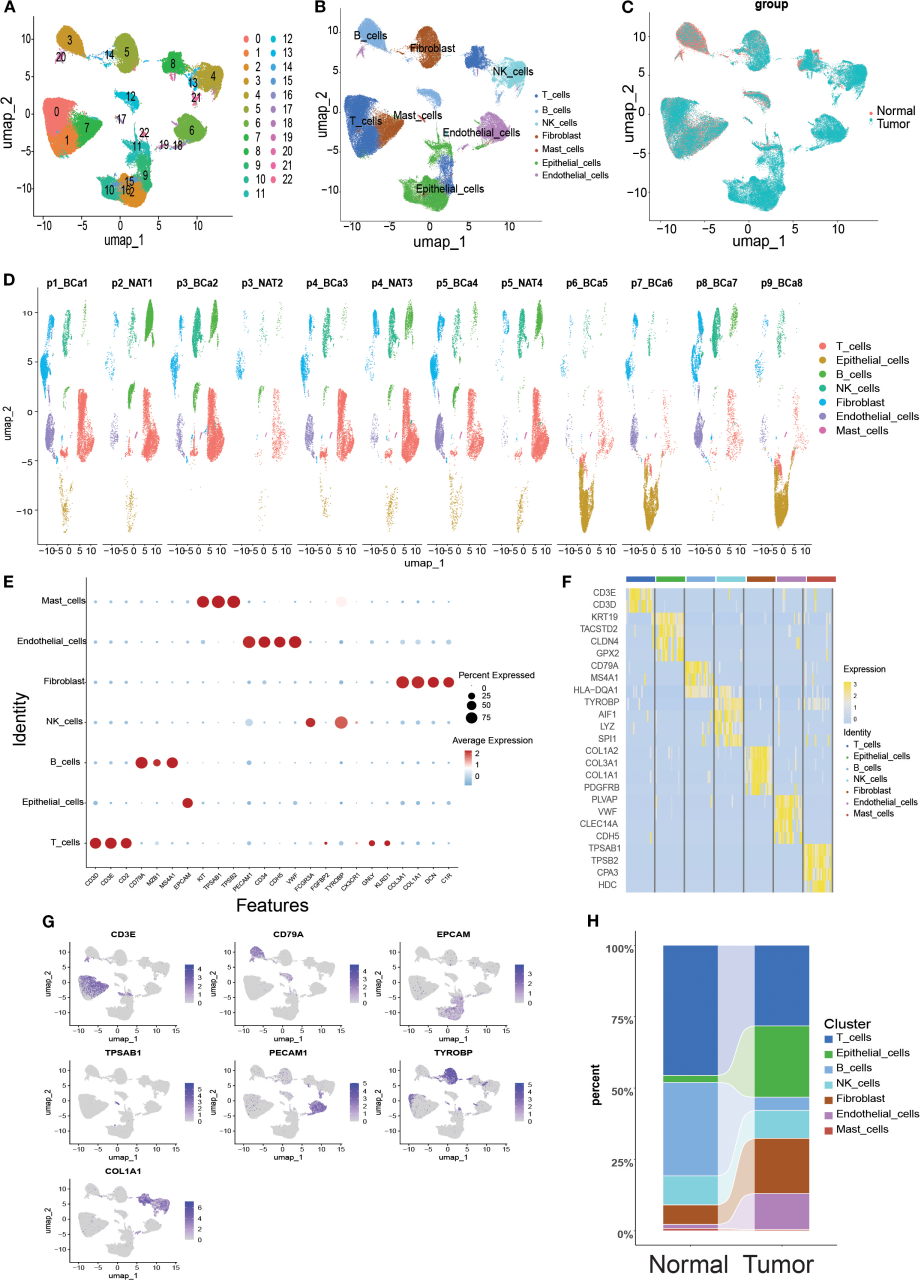
Overview of single-cell mapping of normal and tumor samples from bladder cancer cancer patients. **(A)** Umap plot depicting single cell samples clustered into 23 clusters. **(B)** Identification of 7 cell types based on marker gene expression. **(C)** Umap clustering diagram comparing normal and tumor tissues. **(D)** Umap clustering diagram of 12 samples. **(E)** Bubble plots showing marker gene expression of 7 cell types across cells. **(F)** Heatmap showing differential gene expression across 7 cell types. **(G)** Umap plot highlighting expression patterns of marker genes across 7 cell types. **(H)** Cell type sections of normal and tumor samples in the scRNA-seq dataset.

Twelve samples (including tumors and paired normal tissues) from nine patients in the GSE222315 dataset were analyzed in this study. After rigorous quality control screening, 84,967 high-quality cells were obtained for subsequent analysis. After eliminating the batch effect using the ‘anchor’ integration strategy, the data were normalized, centered and downscaled by principal component analysis (PCA) to retain the top 30 principal components. Subsequent clustering visualization using the t-SNE algorithm classified the cell population into 23 cell clusters (**Figure 2A**), which were grouped into seven major cell types by manual annotation (**Figure 2B**). **Figure 2C** shows the single-cell transcriptome profiles of the normal and tumor samples. **Figure 2D** shows single-cell transcriptome profiles of different samples using manual cellular annotation revealing seven cell types (**Figure 2E**): *CD3D*, *CD3E*, *CD2*, *GNLY*, *KLRD1* high-expressing T cells, *EPCAM* high-expressing epithelial cells, *CD79A*, *MZB1*, *MS4A1* high-expressing B cells, *FCGR3A*, *TYROBP*-overexpressing NK cells, *COL3A1*, *COL1A1*, *DCN*, *C1R*-overexpressing Fibroblast cells, *PECAM1*, *CD34*, *CDH5*, *VWF*-overexpressing Endothelial cells, and *KIT*, *TPSAB1*, and *TPSB2*-overexpressing Mast cells. Differential gene expression heatmaps (**Figure 2F**) and UMAP maps (**Figure 2G**) were used to visualize the transcriptional features and marker gene expression patterns of the seven cell types. In addition, we analyzed the proportional distribution of each cell type in the normal and tumor tissues (**Figure 2H**).

### 3.2 Single-cell transcriptome mapping of B cells in scRNA-seq

**Figure 3:**
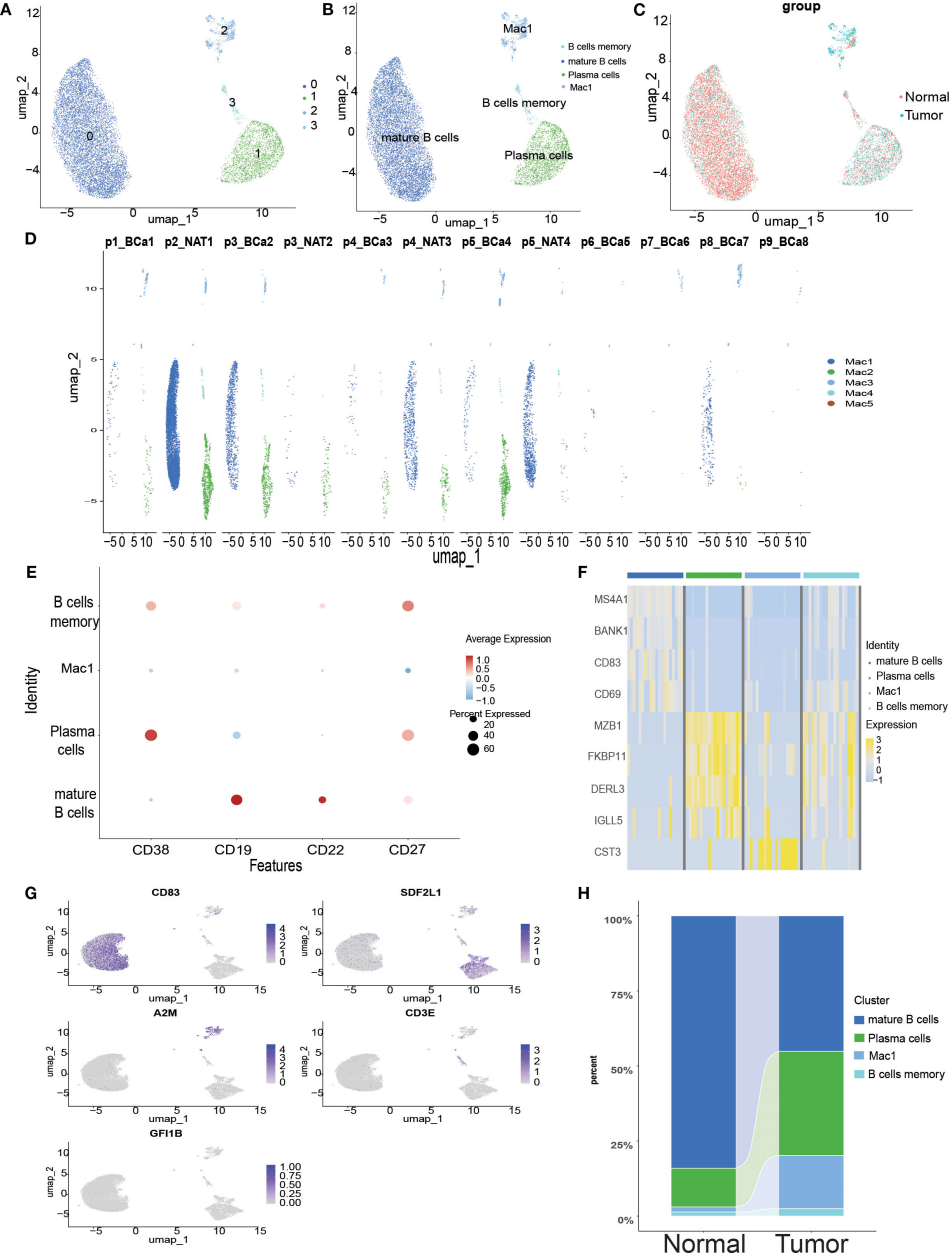
Single-cell transcriptome mapping of B cells in scRNA-seq. **(A)** Umap plot of B cells in scRNA-seq, clustered into 5 different clusters. **(B)** Sequence-based identification of B based on 5 cell types. **(C)** Umap clustering plot comparing normal and tumor tissues. **(D)** Umap clustering plot of 12 samples. **(E)** Bubble map showing marker genes for 5 B-cell subtypes. **(F)** Heatmap showing differential gene expression for 5 cell types. **(G)** Umap plot highlighting the expression patterns of marker genes for the 5 cell types. **(H)** River bar stack plot of cell types for normal and tumor samples in the scRNA-seq dataset.

ScRNA-seq analysis of bladder cancer revealed a high abundance of B cells in all samples. In total, 10,967 B cells were extracted for clustering and annotation. Secondary clustering divided these cells into four clusters (**Figure 3A**), which were manually annotated based on marker gene expression patterns (**Figure 3B**). A comparison of UMAP clustering between normal and tumor tissues revealed significant differences (**Figure 3C**). **Figure 3D** illustrates the transcriptomic features of B cells across samples, which were classified into three identifiable B cell subtypes and one unannotated subtype: plasma cells with high *CD38* expression, mature B cells with high *CD19/CD22* expression, and memory B cells with high *CD27* expression (**Figure 3E**). Further analysis demonstrated that these five B cell subtypes exhibited significant differences in gene expression between normal and tumor tissues (**Figure 3F**), and the UMAP plots visually depicted the expression of marker genes across the subtypes (**Figure 3G**). Overall, this single-cell transcriptomic study provides a comprehensive characterization of abundant B cell populations in the bladder tumor microenvironment, offering insights into their potential roles in tumor immune regulation.

### 3.3 Enrichment analyses associated with B cells

**Figure 4:**
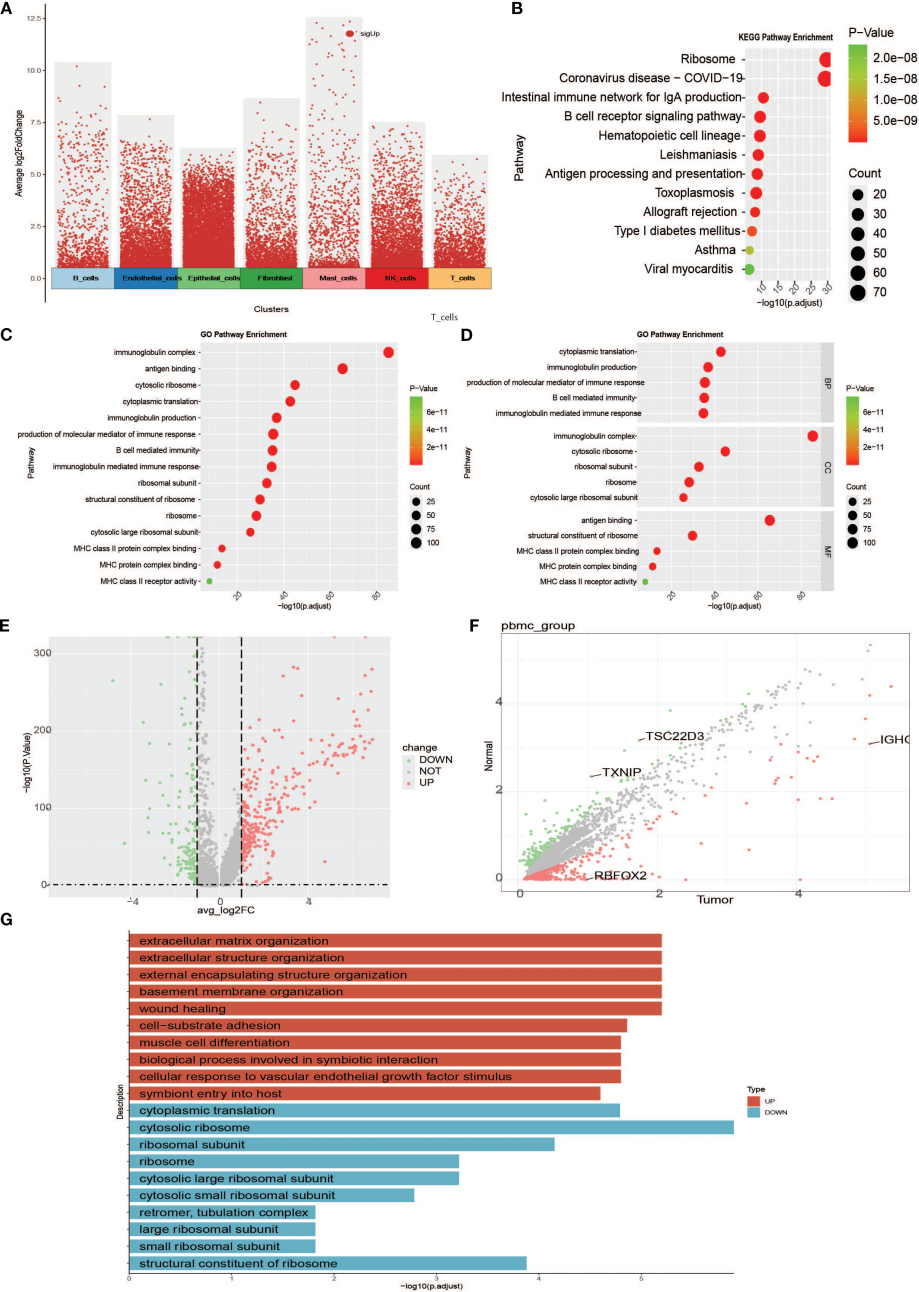
Enrichment analyses between cells of specific subpopulations and between groups. **(A)** Overall cell subpopulation intercellular differential analysis volcano plot (red dots are genes specific to cell clusters relative to other cell clusters). **(B)** B-cell intercellular differential gene KEGG functional enrichment map. **(C)** Functional enrichment of the B cell intercellular differential gene GO. **(D)** B-cell intercellular differential gene GO functional enrichment map (faceted to show the enrichment of BP, MF, and CC in GO entries). **(E)** Scatter plot of B-cell intergroup difference analysis. **(F)** Volcano plot of B-cell intergroup difference analysis. **(G)** Bar graph of differential gene enrichment between B-cell groups.

To investigate specific gene expression among the different cell subtypes, we first performed a volcano plot analysis of the seven cell subtypes (**Figure 4A**). To elucidate the biological functions of the marker genes in B cells, KEGG and GO enrichment analyses were performed. KEGG results showed that marker genes were mainly enriched in pathways activated to function in B cells, including ribosome, coronavirus disease-COVID-19, intestinal immune network for lgA production, B cell receptor signalling pathway, hematopoietic cell lineage, Leishmaniasis, Antigen processing and presentation, Toxoplasmosis, Allograft rejection, and Type I diabetes mellitus (**Figure 4B**). GO enrichment results showed that biological processes (BP) were mainly associated with cytoplasmic translation, immunoglobulin production, production of molecular mediators of immune response, B cell-mediated immunity, and immunoglobulin-mediated immune response. Cellular Component (CC) is mainly related to immunoglobulin complexes, cytosolic ribosomes, ribosomal subunits, ribosomes, and cytosolic large ribosomal subunits. Molecular function (MF) was associated with antigen-binding and structural constituents of ribosomes (**Figures 4C, D**). We also mapped the differential gene scatter plots (**Figure 4E**) and volcano plots (**Figure 4F**) of B cells between tumor and normal tissues and performed differential gene enrichment analyses (**Figure 4G**) to further explore the changing characteristics of B cells in the tumor microenvironment.

### 3.4 pseudotemporal analysis

**Figure 5:**
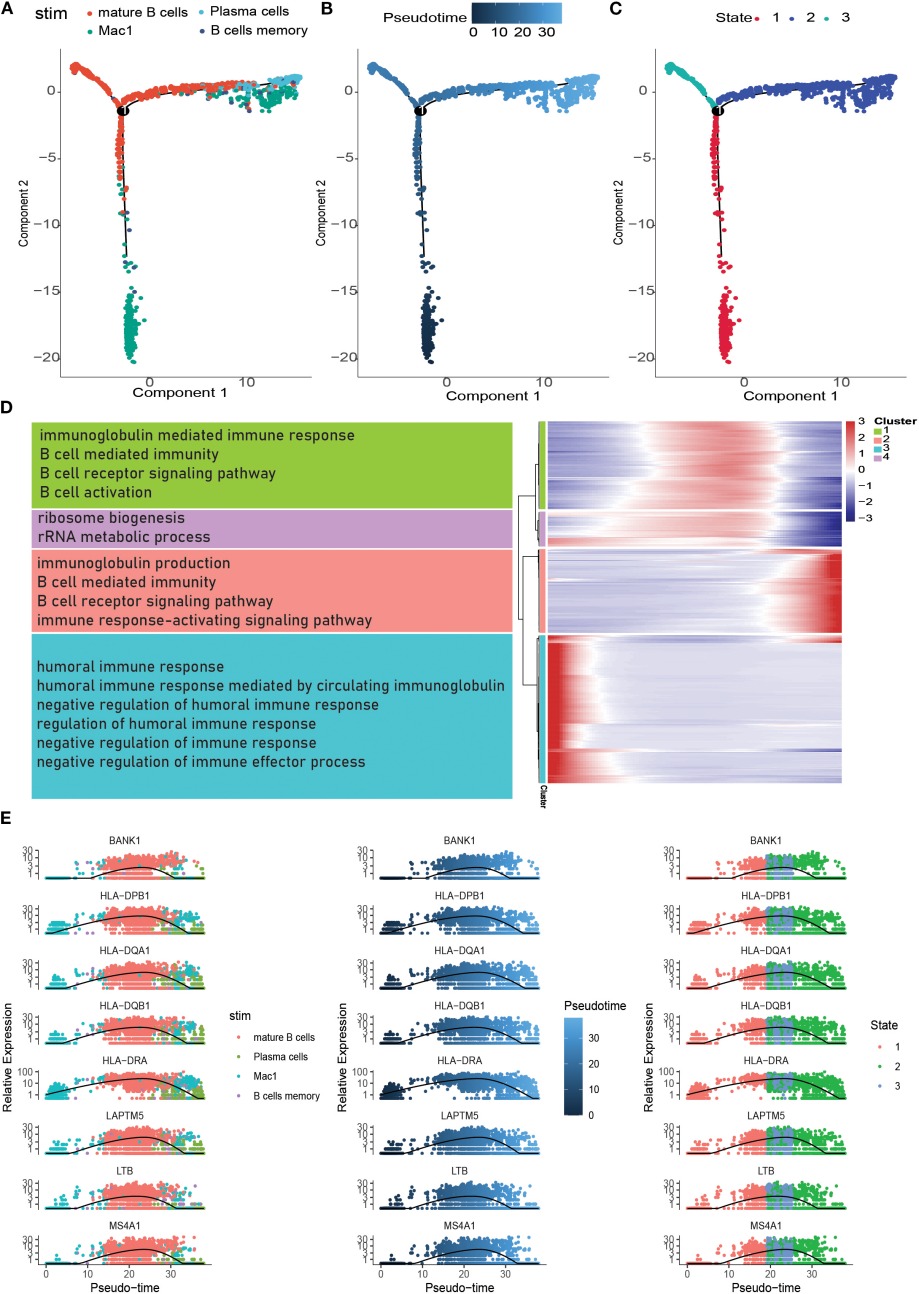
Pseudotime analysis of specific subpopulations. **(A)** Position of different subpopulations in the pseudotime (stim). **(B)** Differentiation of different subpopulations in the pseudotime (Pseudotime). **(C)** Position of cells in different pseudotime stages in the pseudotime (State). **(D)** Heatmap of distribution of genes with differences in the pseudotime as well as the enrichment situation (Heatmap of genes with changes in differences in the pseudotime is shown in four clusters; text in the left bar is the enrichment pathway). **(E)** Scatterplot of gene changes in the pseudotime.

Pseudotemporal analysis, also known as cell trajectory analysis, simulates developmental trajectories of different cells based on the expression patterns of temporal genes in single-cell samples. B-cell maturation trajectories were analyzed in real time using the Monocle software package for tumor-associated B cells. We extracted B cells to demonstrate their developmental trajectories, revealing five branches of B cell subtype (**Figure 5A**). As shown in **Figures 5B, C**, B cells exhibited five differentiation states during development. **Figure 5B** depicts the chronological order of cell subtype differentiation, with darker colored cells gradually transforming into lighter colored ones. The results showed progression from Branch 3 to Branch 5, Branch 1, and Branch 2. There were differences in the expression of model genes at different developmental stages of eutrophication. During the development of tumor-associated B cells, the expression of *DERL3*, *FKBP11*, *FKBP2*, *ITM2C*, *MZB1*, *PRDX4*, *RRBP1*, and *XBP1* gradually increases. Subsequently, the temporal gene expression of the four branches was presented as a heatmap using the BEAM function. BPs were explored by performing GO enrichment analysis, which showed that branch 1 was mainly associated with the immune response-activating signalling pathway, B cell activation, B cell receptor signalling pathway, B cell proliferation, B cell differentiation, and B cell-mediated immunity. Branch 2 is mainly involved in B-cell activation, homeostasis, proliferation, and differentiation. Branch 3 is mainly related to the cellular response to tumor necrosis factor, the response to tumor necrosis factor, and positive regulation of the humoral immune response. Finally, branch 4 was mainly related to the cellular response to tumor necrosis factor, response to tumor necrosis factor, and positive regulation of the humoral immune response (**Figure 5E**). These pathways are mainly involved in B cell differentiation and immune responses.

### 3.5 Analysis of cell-cell interactions associated with B cells

**Figure 6:**
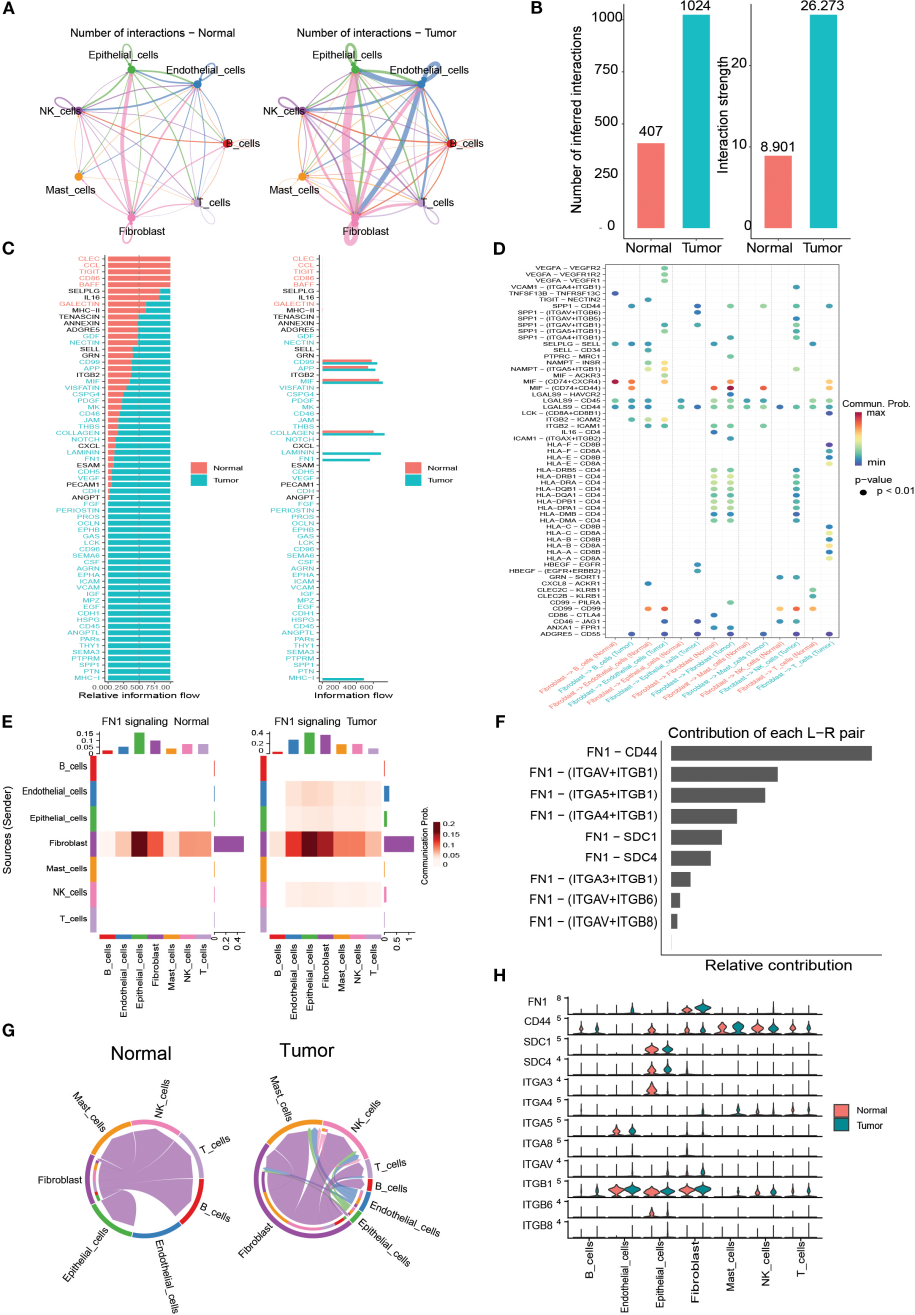
Cell–cell communication analysis. **(A)** Circos plots of intercellular interactions in normal and tumor groups. **(B)** Comparison of the number and strength of cell–cell communications between tumor and normal tissues. **(C)** Differential signaling pathway-related communications between tumor and normal tissues. **(D)** Communication probabilities of macrophages with other cell types mediated by ligand–receptor pairs. **(E)** Heatmap of *FN1* signaling interactions among different cell types in tumor and normal groups. **(F)** Relative contribution of ligand–receptor pairs within the *FN1* pathway in tumors. **(G)** Cell–cell communication mediated by the *FN1*–*CD44* ligand–receptor pair. **(H)** Expressiondifferences of *FN1* pathway molecules between tumor and normal samples.

To explore the communication properties of B cells with other cell types, the Cellchat function was used to detect ligand-receptor and molecular interactions between different cells. The results showed that the interactions between the seven cell types (T cells, B cells, NK cells, fibroblast cells, endothelial cells, and mast cells) were stronger in bladder cancer tissues than in normal tissues (**Figures 6A, B**). The *FN1* signalling pathway was upregulated in tumor samples and was widely present in ligand-receptor interactions in various cell types (**Figures 6C-E**). Among these, the ligand-receptor pair *FN1-CD44* contributed the most to the *FN1* pathway (**Figure 6F**), and *SCD1* and *ITGB1* showed higher expression levels in multiple cell types (**Figures 6G, H**).

### 3.6 Identification of the hub gene in bladder cancer

**Figure 7:**
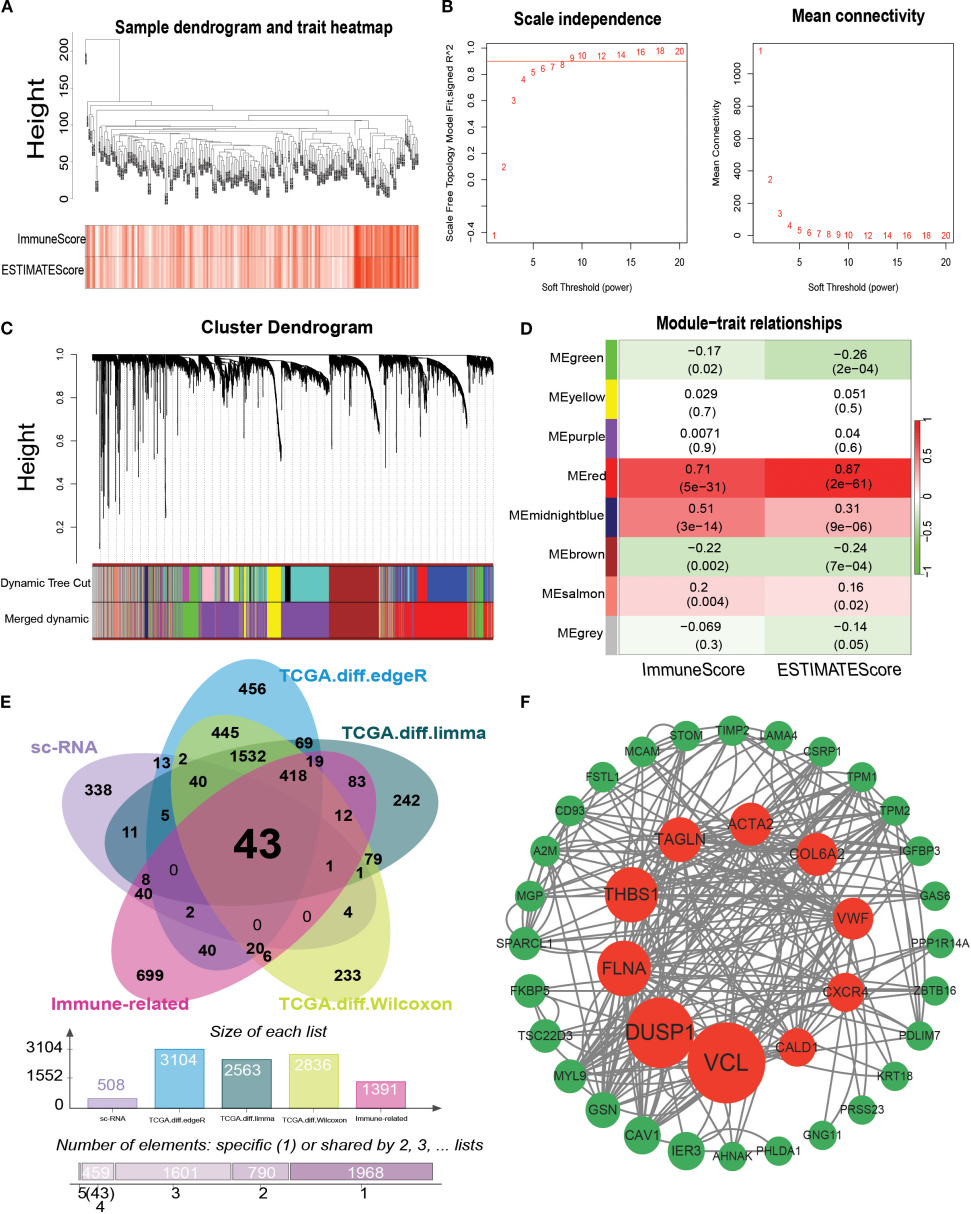
Identification of modules associated with bladder cancer using WGCNA. **(A)** Sample clustering plot; **(B)** Scale-free index and average connectivity analysis for different soft threshold powers. **(C)** Dendrogram of all differentially expressed genes clustered according to the heterogeneity measure (1-TOM). Ribbons show results from automated single-block analysis; **(D)** Heatmap of correlation between module signature genes and bladder cancer immune-related traits. **(E)** Venn plot showing the results of the intersection analysis of b-cell related differentially expressed genes in single cell samples and bladder cancer TCGA dataset, showing a total of 43 immune-related bladder cancer b-cell key genes. **(F)** Protein interactions network graph constructed by the identified 43 key genes, in which the red nodes marked the core genes with the top ten BC values. TOM: topological overlap matrix, ME: module eigengene; BC: betweenness centrality.

WGCNA-based identification of infiltrative immune cell-related genes to screen infiltrative immune cell-related genes, we performed WGCNA assays on the GSE13507 dataset. First, sample clustering analysis showed that all 197 samples in the GSE13507 dataset could be used to construct weighted gene co-expression networks (**Figure 7A**). Subsequently, a weighted gene co-expression network containing eight modules was constructed when *β* was set to 5 (R^2^ = 0.85), and modules with module feature gene dissimilarity less than 0.3 were merged (**Figures 7B, C**). The associations between these eight modules and the four algorithms (StromalScore, ImmuneScore, ESTIMATEScore, and TumorPurity) are shown in **Figure 7D**. We found that the MEred module was associated with the ImmuneScore (**Figure 7D**). In addition, we calculated the correlation between the genes in the MEred module and ImmuneScore and identified 1390 infiltrating immune cell-associated genes by setting the correlation coefficient.

Gene expression datasets of 412 bladder cancer tissues and 19 paracancerous tissues were obtained from TCGA database. Differential expression analysis between bladder cancer and paracancerous tissues was performed to screen for genes related to bladder carcinogenesis and progression. Three methods, R package DESeq, limma, and Wilcoxon, were used to identify DEGs, and the screening criteria were set as |log2 fold change | > 2 and a corrected *P* < 0.05. A total of 3104, 2563, and 2836 differentially expressed genes were identified using these three methods, respectively (**Figure 7E**). This study investigated bladder cancer B-cell marker genes, immune-related genes screened using WGCNA, and bladder cancer-related genes obtained by differential analysis (**Figure 7E**). A total of 43 shared genes were identified. To explore the interaction network of core genes at the protein level, the PPI network was constructed using the STRING database and visualized using Cytoscape 3.7.1 (**Figure 7F**) Ten core genes were obtained by screening based on BC values: *VCL*, *DUSP1*, *FLNA*, *THBS1*, *TAGLN*, *ACTA2*, *COL6A2*, *VWF*, *CXCR4*, and *CALD1*.

### 3.7 Evaluating the prognostic and diagnostic value of key genes

**Figure 8:**
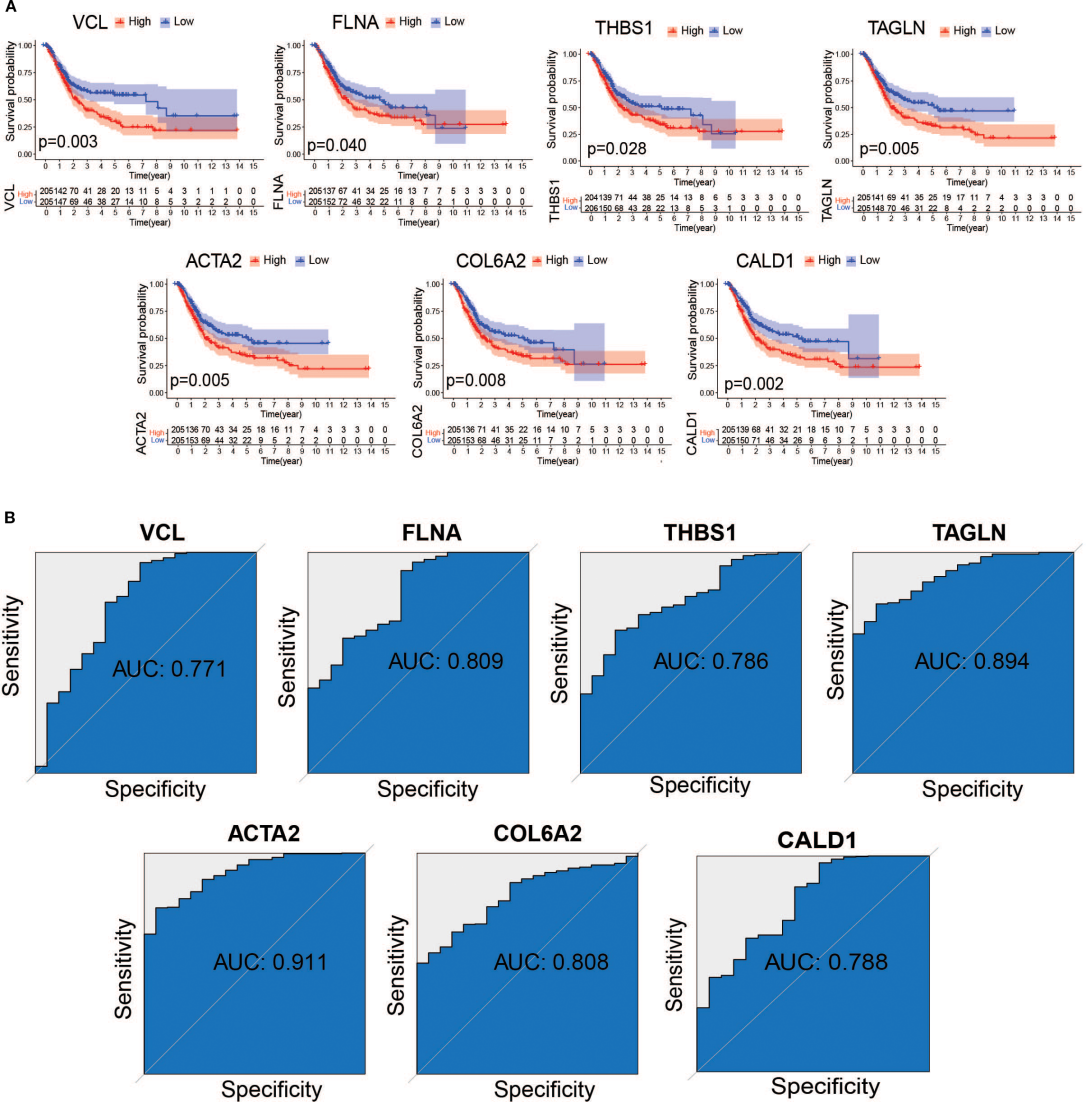
Assessment of the value of hub gene diagnosis. **(A)** Survival curves of 8 genes with prognostic value **(B)** Receiver Operating Characteristic (ROC) curves of hub genes in the TCGA dataset.

To assess the association between core genes and bladder cancer prognosis, we systematically evaluated ten candidate genes using Kaplan-Meier survival analysis combined with the log-rank test. The results of the analysis showed that seven genes (*VCL*, *FLNA*, *THBS1*, *TAGLN*, *ACTA2*, *COL6A2*, and *CALD1*) were significantly correlated (*P* < 0.05) with the prognosis of bladder cancer patients, and these genes were included in the subsequent in-depth study (**Figure 8A**). To further validate the diagnostic value of these core genes in distinguishing between rejected and non-rejected samples, we performed subject work characteristic (ROC) curve analyses in the TCGA training and GSE37815 validation sets. The results showed that the area under the curve (AUC) for all seven core genes exceeded 0.7 in both independent datasets (**Figure 8B**), strongly suggesting that these genes may serve as potential biomarkers for bladder cancer diagnosis.

### 3.8 Key gene database and protein expression results

**Figure 9:**
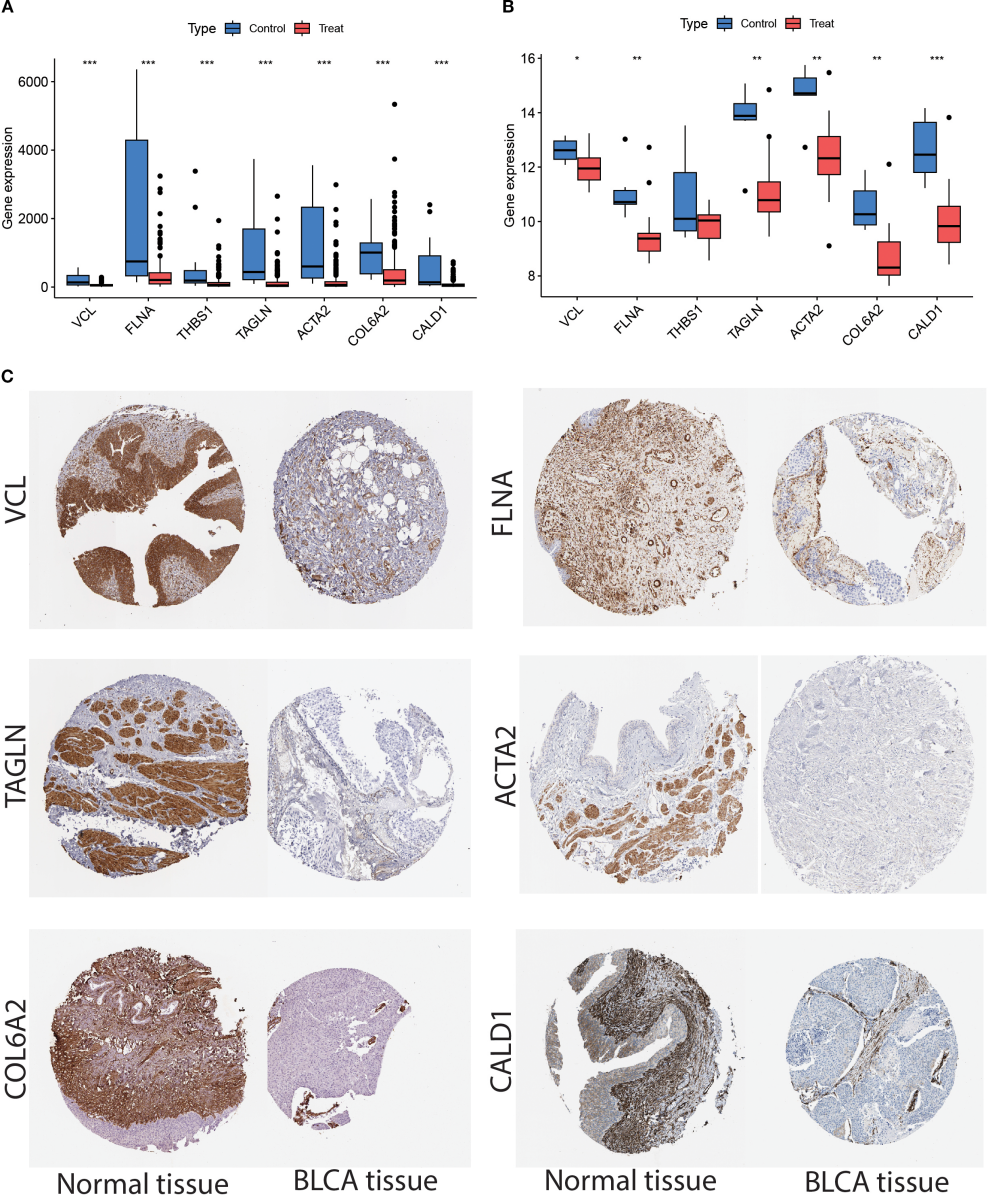
**(A)** TCGA dataset showed that the expression of *VCL*, *FLNA*, *THBS1*, *TAGLN*, *ACTA2*, *COL6A2*, and *CALD1* were down-regulated in the tumor samples as compared to normal samples. **(B)** Analysis of the GSE37815 dataset revealed that *VCL*, *FLNA*, *TAGLN*, *ACTA2*, *COL6A2*, and *CALD1* was downregulated in tumor samples compared to normal tissues, whereas *THBS1* showed no significant difference. The Wilcoxon rank-sum test was used to assess discrepancies between the different samples. Each p-value is written above the box plots (NS: p > 0.05, *: p < 0.05, **: p < 0.01, ***: p < 0.001, and ****: p < 0.0001). **(C)** Validation of the expression of the six genes in the Human Protein Atlas (HPA) database showed that their expression levels were significantly lower in the tumor samples than in the normal group.

We then performed an in-depth analysis of the expression patterns of these key genes in the TCGA and GSE37815 datasets, focusing on comparing the expression differences between tumor tissues and paired non-tumor tissue samples. The results showed that, except for *THBS1*, the remaining six genes exhibited statistically significant differential expression in both the training and validation sets (*P* < 0.05); therefore, *THBS1* was excluded from subsequent analyses (**Figures 9A, B**). Ultimately, six genes—*VCL*, *FLNA*, *TAGLN*, *ACTA2*, *COL6A2*, and *CALD1*—were identified as potential marker genes. To investigate protein-level interactions between these six candidate markers and *FN1*, a protein-protein interaction (PPI) network was constructed. As shown in **Supplementary Figure 1**, *FN1* displayed strong interactions with all the six marker proteins. As shown in **Figure 9C**, compared to normal tissues, *VCL*, *FLNA*, *TAGLN*, *ACTA2*, *COL6A2*, and *CALD1* were significantly downregulated in BLCA tissues.

### 3.9 RT-qPCR results of key genes in cell lines and clinical samples

**Figure 10:**
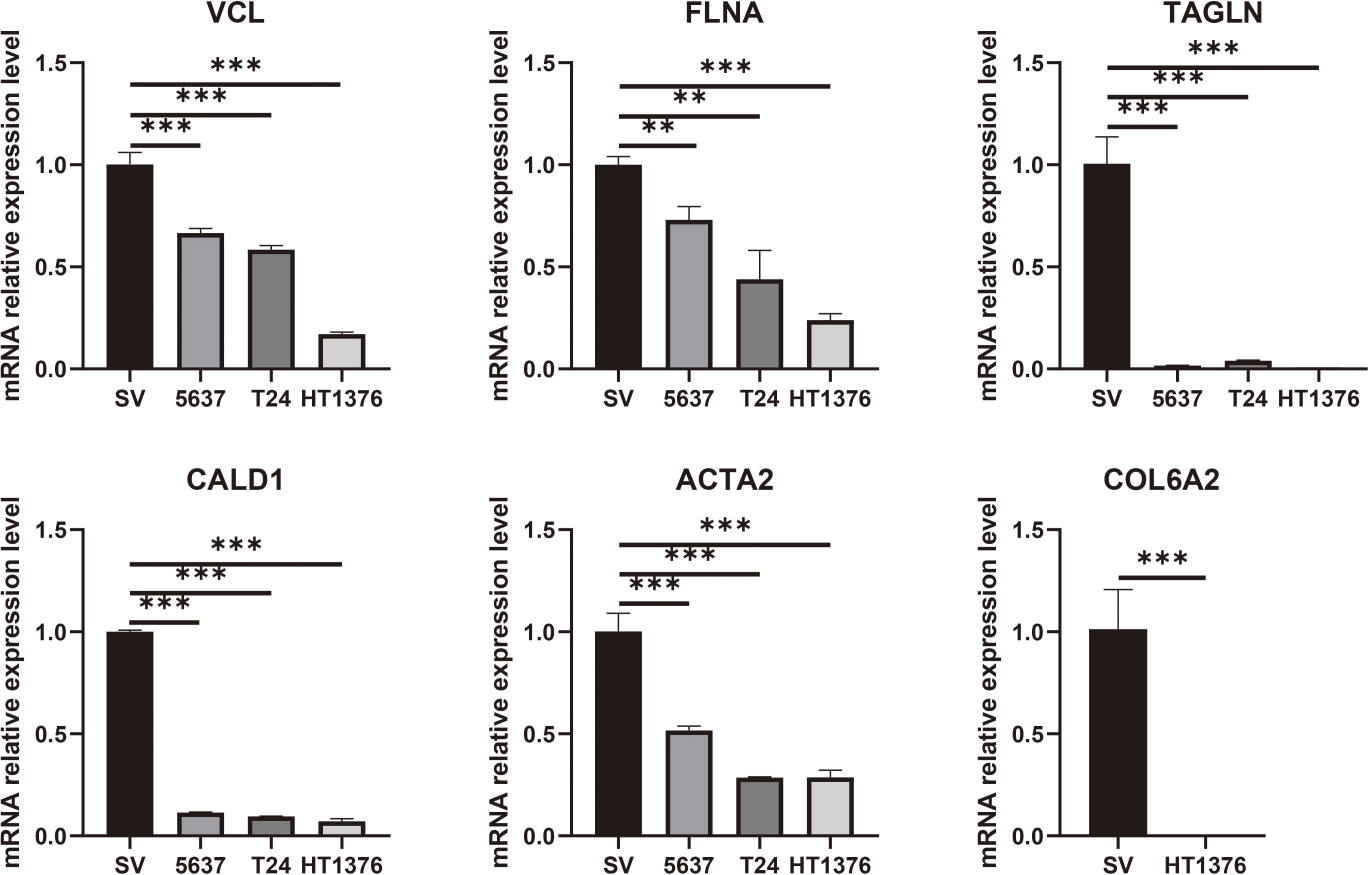
RT-qPCR validation of six key genes (*VCL*, *FLNA*, *TAGLN*, *CALD1*, *ACTA2*, and *COL6A2*) was performed in cell lines. The results showed that these genes were significantly downregulated in bladder cancer cells. Statistical significance is indicated above the bars in the histogram (NS: p > 0.05, *: p < 0.05, **: p < 0.01, ***: p < 0.001, and ****: p < 0.0001).

**Figure 11:**
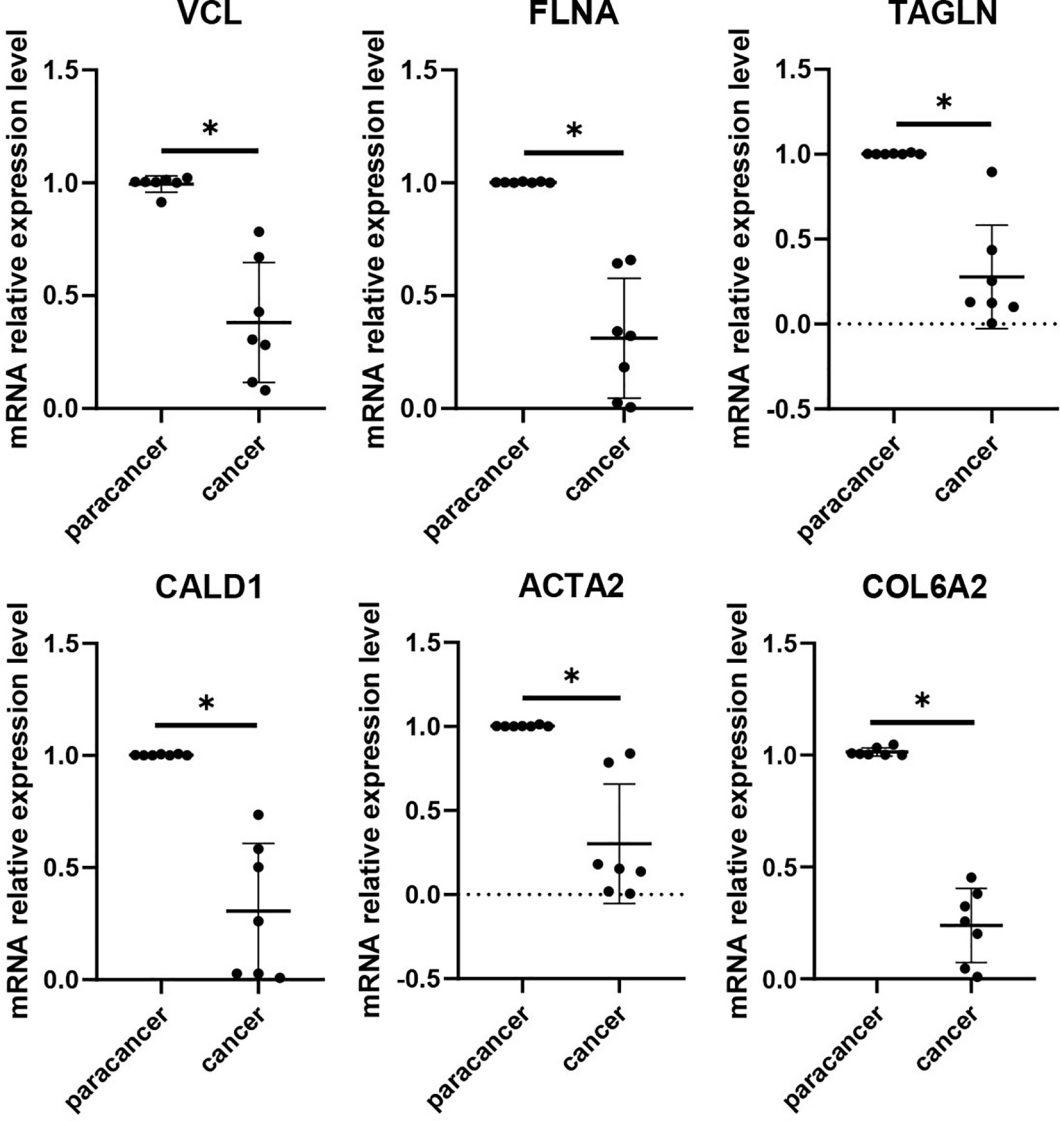
RT-qPCR validation of six key genes (*VCL*, *FLNA*, *TAGLN*, *CALD1*, *ACTA2*, and *COL6A2*) was performed in tumor tissues and adjacent normal tissues from bladder cancer patients. The histogram shows that all six genes were significantly downregulated in tumor tissues. Differences between tumor and adjacent normal tissues were assessed using the Wilcoxon rank-sum test, and statistical significance is indicated above the bars in the histogram. (NS:p > 0.05;:p < 0.05;:p < 0.01;:p < 0.001;****:p < 0.0001).

To validate the expression differences of the six potential biomarkers, further experiments were performed on bladder cancer cells and normal bladder cells. RT-qPCR results showed that compared with normal bladder epithelial cells SV-HUC-1, the expression levels of six biomarkers, namely *VCL*, *FLNA*, *TAGLN*, *ACTA2*, *COL6A2* and *CALD1*, the expression levels of all six biomarkers were significantly down-regulated (*P* < 0.05), a result that was highly consistent with the results of our pre-biomarker analysis (**Figure 10**). Further validation was performed in tumor tissues and adjacent normal tissues from seven bladder cancer patients, and the results showed that the expression changes of the six downregulated genes were consistent with bioinformatics analysis and cell experiments (**Figure 11**). Overall, Alterations in the expression of *VCL*, *FLNA*, *TAGLN*, *ACTA2*, *COL6A2*, and *CALD1* may play important roles in the development of bladder cancer, suggesting their potential value in early detection, molecular subtyping, and targeted therapy.

## 4 Discussions

Despite advances in preclinical and clinical studies, the prognosis of bladder cancer remains unclear. scRNA-seq technology can reveal cellular features at single-cell resolution and elucidate the regulatory mechanisms of RNA expression in pathological states (30, 31), offering the possibility of accurately identifying prognostic features associated with germinal centres. As important participants in the antitumor immune response, B cells play a key role in the presentation of tumor antigens to T cells (32). In this study, we explored the B cell-associated differentially expressed gene profiles based on scRNA-seq technology and preliminarily explored the potential clinical application value of these genes in early bladder cancer screening and molecularly targeted therapies by integrating TCGA and GEO datasets.

In this study, 52,721 single-cell transcriptomes from bladder cancer and normal samples were analyzed to construct a complete map of the TME of bladder cancer. Seven cell types were identified by annotation: T cells, B cells, endothelial cells, epithelial cells, fibroblasts, NK cells, and mast cells. Cell type identification revealed the presence of 10,967 B cells. Notably, the analysis showed that the expression level of B cells was significantly lower in tumor tissues than in normal tissues. It has been shown that B cells perform differentiated functions in different cancer types (33). B cells secrete immunoglobulins, which in turn inhibit tumor growth (34). Kroeger et al. found clonal B-cell expansion and increased plasmoblastoid infiltration in the tumor tissues of patients with bladder cancer, and B cells, as antigen-presenting cells, activated tumor-specific T-cell responses (35). These studies revealed an immune-mediated relationship between B cells and the pathogenesis of bladder cancer and that downregulation of B cell expression in the tumor microenvironment may lead to a reduction in the production of immune proteins, which in turn exacerbates tumor progression. The association between downregulated B cell expression in bladder cancer and increased bladder cancer risk observed in this study is consistent with the above findings. This study provides new insights into the future development of tumor immunotherapy and highlights the importance of fully considering the role of B cells in tumors.

In addition, GO enrichment analysis showed that B cell marker genes were significantly enriched in five immune-related pathways: immunoglobulin production, production of molecular mediators of the immune response, B cell-mediated immunity, B cell-mediated immunity, immunoglobulin-mediated immune response, and immunoglobulin complex. KEGG results showed that marker genes were significantly enriched in the B-cell receptor signalling pathway, a pathway in which B cells are activated to function. B-cell pseudotemporal trajectory analyses revealed dynamic changes in gene expression over time. These genes were classified into four functional clusters involved in lymphocyte-mediated immunity, protein folding, immunoglobulin, and cytoplasmic translation processes. Intercellular communication network analysis revealed a close association between ligands and receptors in different cells, in which cytokines mainly interact through the *FN1* (Fibronectin 1) signalling pathway, a finding that highlights the value of *FN1* as a potential biomarker for bladder cancer. Heat map analysis further showed that the expression of genes related to the *FN1* signalling pathway was mainly reflected in the interaction between fibroblasts and epithelial cells.

Evidence suggests that *FN1* positively regulates the proliferation and migration of a variety of cancer cells and has been identified as a biomarker for a number of cancers, including gastric cancer (36) and cervical cancer (37). Targeted inhibition significantly reduces the proliferation, invasive ability, and metastatic potential of cancer cells. Zhang et al. revealed that *FN1* may be involved in the development and progression of bladder cancer and has potential as a prognostic marker and therapeutic target for bladder cancer (38). In this study, we confirmed the critical role of *FN1* in bladder cancer through in-depth analyses, identified it as a potential oncogenic pathway in bladder cancer, and elucidated its functional mechanism in fibroblasts. Additionally, our study found that FN1 exhibits strong protein-protein interactions with six proteins, including *VCL*, *FLNA*, *TAGLN*, *ACTA2*, *COL6A2*, and *CALD1*. Previous studies have also reported an interaction between *FN1* and *VCL*, consistent with our findings (39). Based on the above findings, the protein-protein interactions between *FN1* and these six identified biomarkers require further experimental validation. These findings not only open a new direction for *FN1*-targeted therapy but also show important research and clinical application prospects in enhancing the effectiveness of cancer immunotherapy and deepening the knowledge of the dynamic role and molecular mechanisms of *FN1* in cancer.

Next, the TCGA bladder cancer dataset was analyzed for differences. Subsequently, immune-related genes were screened using WGCNA in the GEO dataset. Finally, six B-cell marker genes (*VCL*, *FLNA*, *TAGLN*, *ACTA2*, *COL6A2*, and *CALD1*) that were downregulated in bladder cancer were screened using the PPI network, survival curves, ROC curve analysis, and expression validation. Previous studies have shown that Vinculin (*VCL)* expression is downregulated in bladder cancer with tumor suppressor gene properties (40), which is consistent with the results of the present study. Wu et al. further confirmed that *VCL* can be used as a potential protein marker for bladder cancer (41) and provided strong support for the results of this study. Filament protein A (*FLNA*) plays a key role in the development of blood vessels, heart, and brain organs, particularly in the formation of intercellular contacts and adherent junctions (42). It has been demonstrated that the expression level of *FLNA* is significantly reduced in bladder cancer tissues and that *FLNA* overexpression can effectively inhibit the proliferation, invasion, and metastasis of bladder cancer cells (43), corroborating the results of the present study. Transglutamine protein (*TAGLN*) is an important actin-associated protein, which has been found to be expressed at significantly reduced levels in a variety of cancers, including prostate cancer. Based on this feature, *TAGLN* is widely considered to have tumor suppressor effects (44), and further studies have shown that *TAGLN* may not only serve as a potential diagnostic biomarker for bladder cancer (BCa), but may also be a promising therapeutic target (45). The actin alpha 2 (*ACTA2*, actin alpha 2) gene is primarily responsible for encoding smooth muscle alpha-actin, an essential component of the cytoskeleton, which plays a central role in key biological processes, such as cell contraction, migration, and signal transduction (46). Previous studies have shown that *ACTA2* is one of the pivotal genes closely associated with the immune response in muscle invasive bladder cancer (MIBC) (47), which not only reveals the important role of *ACTA2* in the development of MIBC but also provides a new research direction for an in-depth understanding of the mechanism of tumor immune microenvironment regulation. The collagen type VI alpha 2 chain (*COL6A2*) gene is an important component of the three alpha chains encoding collagen type VI. Recent studies have shown that *COL6A2* plays a key role in many tumors, especially lung adenocarcinoma, and its expression level can be used as a prognostic predictor and a potential marker for targeted therapies (48). Notably, through an in-depth study of bladder cancer tissues, Piao et al. found that the mRNA expression level of *COL6A2* was significantly downregulated in both non-muscle-invasive bladder cancer (NMIBC) and muscle-invasive bladder cancer (MIBC) tissue samples compared to that in normal bladder tissues (49), which is consistent with our findings. Caldesmon 1 (*CALD1*) is an important cytoskeleton-associated protein that affects cell morphology and migration capacity by regulating the dynamic balance of actin filaments (50). Recent studies have revealed that *CALD1* not only serves as a prognostic biomarker for bladder cancer but may also contribute to its progression of bladder cancer by participating in the remodelling process of the tumor microenvironment. Further studies have shown that the expression of *CALD1* is significantly correlated with immune cell infiltration in bladder cancer, which provides a new perspective for understanding the regulation of the tumor immune microenvironment (51).

Although the detection of downregulated genes in clinical practice presents certain challenges, they can still serve as reliable biomarkers, and their clinical feasibility has been supported by multiple lines of evidence. First, the principle of “loss indicates abnormality” suggests that the reduced expression of tumor suppressor genes itself can indicate pathological changes. This mechanism is exemplified by classical tumor suppressors such as *TP53*, the “guardian of the genome,” which regulates the cell cycle, DNA damage repair, and apoptosis (52), and whose dysfunction or downregulation is implicated in over 50% of human tumors (53). Although the downregulated genes in our study were not classical tumor suppressors, their reduced expression may similarly contribute to bladder cancer development, supporting their potential as diagnostic biomarkers or therapeutic targets. Consistently, previous studies have shown that downregulated genes can serve as prognostic biomarkers and immunotherapy targets. For example, *SOX7* is downregulated in colorectal cancer, and its low expression correlates with poor prognosis (54). Second, the detection of downregulated genes can be achieved through several approaches, including: (1) highly sensitive RNA quantification techniques (e.g., RT-qPCR), which allow accurate discrimination even at low expression levels; (2) protein-level assessment of functional loss, such as immunohistochemistry (IHC) detection of *PTEN* loss, which is widely used as a prognostic indicator in prostate cancer (55, 56); and (3) epigenetic markers such as DNA methylation, which provide stable signals, exemplified by the FDA-approved colorectal cancer screening test Epi proColon^®^, based on *SEPT9* promoter methylation (57). Collectively, these findings suggest that although downregulation itself may be difficult to capture directly, its associated transcriptomic, proteomic, and epigenetic alterations can be transformed into stable and detectable biological signals. Therefore, the six genes identified in our study as negatively associated with bladder tumors may represent promising diagnostic biomarkers and potential therapeutic targets for clinical application.

This study has made significant progress in constructing a B cell-based prognostic model for bladder cancer and its tumor microenvironment. However, this study had limitations. First, although the B cell prognostic model developed using public datasets demonstrated good predictive performance, its clinical utility requires validation through large-scale prospective clinical studies. Second, due to limitations in data availability, the sample size included in this study was relatively small; future multi-center, large-cohort studies are needed to further assess the robustness of the model. Finally, although we confirmed the expression levels of key prognostic genes and their associations with patient outcomes using public clinical data and RT-qPCR experiments, in-depth functional studies on these genes have not yet been conducted. Therefore, in future studies, we plan to employ systematic gene editing and functional assays to elucidate the molecular mechanisms of these key genes in bladder cancer progression, providing a stronger theoretical foundation for clinical applications.

## 5 Conclusion

In this study, we constructed single-cell transcriptome profiles of bladder cancer using scRNA-seq, and explored the role of B cells in cell trajectories, transcription factor regulatory networks, and intercellular communication mechanisms. By integrating single-cell samples with TCGA and GEO data, six immune cell-associated genes closely related to B cells, with significant differences, were successfully identified. These findings suggest six potential molecular targets for bladder cancer treatment. Although the specific mechanisms of action of these genes need to be validated by further studies, these results are important for an in-depth understanding of the molecular mechanisms of bladder cancer pathogenesis.

## Data Availability

The original contributions presented in the study are included in the article/**Supplementary Material**, further inquiries can be directed to the corresponding author/s.
