# Supplementary material for: Comprehensive analysis of single-cell and bulk transcriptomes reveals key B-cell genes and immune microenvironment regulation in bladder cancer
Source: Front Immunol. 2025 Oct 17;16:1600254. doi: 10.3389/fimmu.2025.1600254 (PMC12575231; doi:10.3389/fimmu.2025.1600254)
Supplement: Supplementary file 1 [file DataSheet1.docx]

Supplementary Material

# Supplementary Figures

Figure S1: Protein-protein interaction (PPI) network diagram of FN1 and six biomarkers.

## Supplementary Figures


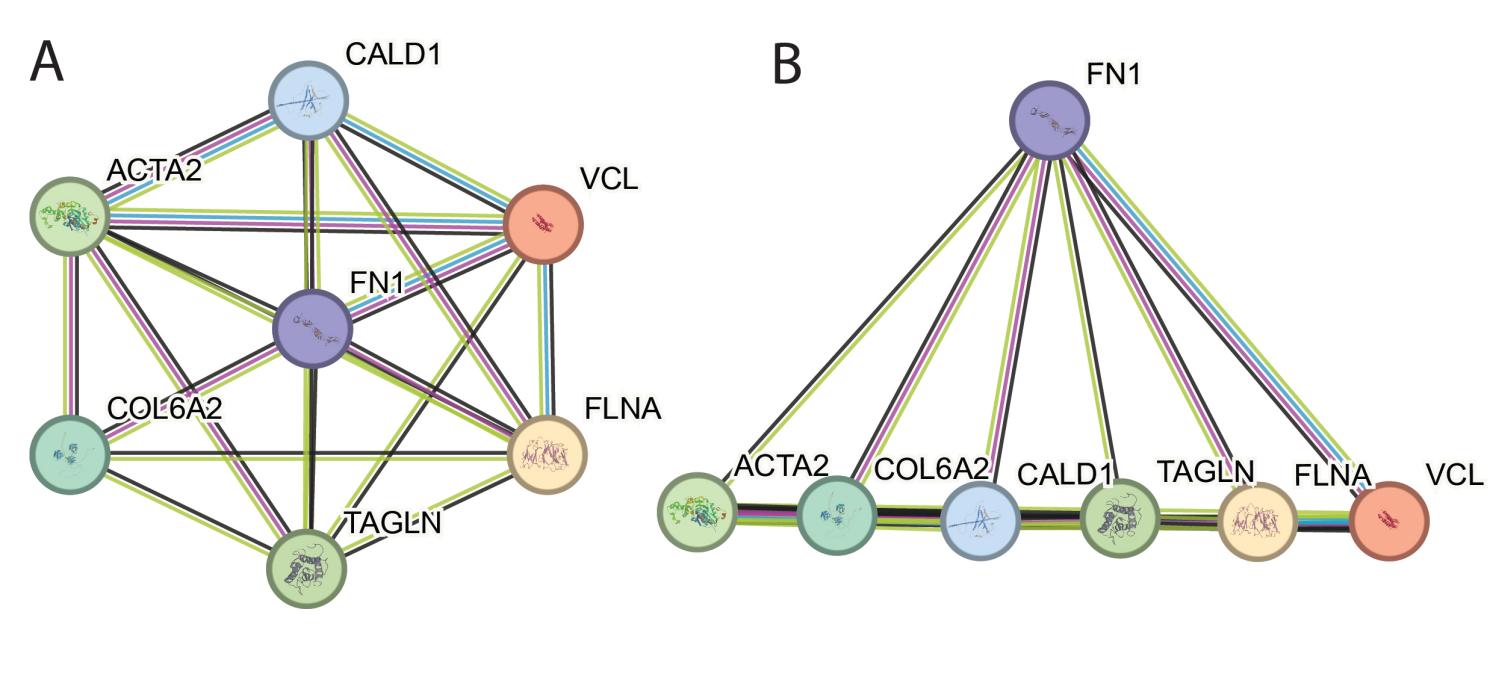


**Supplementary Figure S1.** (A-B) Protein-protein interaction (PPI) network diagram of FN1 and six biomarkers
